# *Ganoderma microsporum* Immunomodulatory Protein (GMI) Enhances Phagocytosis by Suppressing STAT3/CD47 Signaling in EGFR-Mutant NSCLC Resistant to Gefitinib and Osimertinib

**DOI:** 10.7150/jca.124363

**Published:** 2026-01-01

**Authors:** Ya-Chu Hsieh, I-Lun Hsin, Ling-Yen Chiu, Yu-Chien Hung, Yu-Ting Kang, Hui-Yi Chang, Ching-Hsiung Lin, Jiunn-Liang Ko, Yu-Fan Liu

**Affiliations:** 1 Institute of Medicine, Chung Shan Medical University, Taichung 402, Taiwan.; 2 Department of Ophthalmology, Chung Shan Medical University Hospital, Taichung, Taiwan.; 3 Institute of Medicine, Chung Shan Medical University, Taichung, Taiwan.; 4 Department of Medical Research, Taichung Veterans General Hospital, Taichung, Taiwan.; 5 Division of Chest Medicine, Department of Internal Medicine, Changhua Christian Hospital, Changhua City, Taiwan.; 6 Divisions of Medical Oncology and Pulmonary Medicine, Department of Internal Medicine, Chung Shan Medical University Hospital, Taichung 402, Taiwan.; 7 Department of Biomedical Sciences, Chung Shan Medical University, Taichung, Taiwan.; 8 Division of Allergy, Department of Pediatrics, Chung Shan Medical University Hospital, Taichung, Taiwan.

**Keywords:** Ganoderma immunomodulatory protein, Epidermal growth factor receptor, Signal transducer and activator of transcription 3, CD47, Phagocytotic, Lung cancer.

## Abstract

Target therapy is effective for epidermal growth factor receptor (EGFR) mutation in non-small cell lung cancer (NSCLC). However, resistance often occurs after treatment for several months. Macrophages have difficulty in devouring resistant cells. *Ganoderma* immunomodulatory protein (GMI) exhibits anti-tumour and immunomodulatory activities. This study aimed to investigate whether GMI overcomes Osimertinib (Tagrisso) and Gefitinib (Iressa) resistance via enhancing macrophage polarization. GMI attenuated signal transducer and activator of transcription 3 (STAT3) phosphorylation and downstream CD47 expression in parental and resistant cells via Western blot and RT-qPCR. Overexpressed STAT3 restored GMI-induced apoptosis and GMI-reduced transcription of CD47 in HCC827 and H1975 lung cancer cells. Phospho-STAT3 inhibitor (W1131) also reduced the expression of CD47 in NSCLC cells. The interaction between GMI and W1131 was effective in reducing phosphorylated STAT3 and CD47. ImageXpress Pico analysis revealed that GMI enhanced phagocytotic activity of macrophages toward tumour cells with Red CMTPX and Green CMFDA dyes. The results showed that GMI enhanced macrophage phagocytosis of lung cancer cells by inhibiting STAT3 and reducing CD47 expression. In addition, GMI enhanced M1 inhibition of M2 polarization but had no effect on M1 differentiation. This is the first study to demonstrate that GMI enhances macrophage phagocytosis and modulates the STAT3-CD47 axis to overcome EGFR-TKI resistance in NSCLC, highlighting its potential as a novel adjunct immunotherapeutic agent.

## 1. Introduction

Lung cancer remains the first most prevalent human malignancy and represents a leading cause of cancer-related deaths worldwide in the latest GLOBOCAN estimates 1. Several phase III clinical trials have established that gefitinib and erlotinib (first-generation tyrosine kinase inhibitors [TKIs]), as well as afatinib (a second-generation TKI), significantly outperform conventional chemotherapy in terms of progression-free survival and overall response rates for patients with EGFR mutation 2. These mutated epidermal growth factor receptors are recognised as well-known biomarkers for targeted therapy. Despite the high response rate of first-generation TKIs such as gefitinib, most patients will experience disease progression after 9-13 months of treatment 3, 4. This situation has been described as 'acquired resistance' to TKIs.

The most common resistance mechanism to first-generation TKIs is the p.Thr790Met point mutation (T790 M), accounting for almost 50% 5. Osimertinib, an irreversible third-generation TKI designed to overcome resistance to T790M, also covers sensitising EGFR mutations (19Del and 21L858R) 6. There is an urgent need to understand the mechanisms underlying resistance to Osimertinib.

Fungal immunomodulatory proteins (FIPs) are a family of peptides that can regulate immunity, fight inflammation, fight allergies and fight cancer. To date, more than 38 types of FIPs have been discovered 7. *Ganoderma microsporum* immunomodulatory protein (GMI) belongs to the family of FIPs. Previous studies have also confirmed that GMI has various anti-inflammatory, tumour metastasis-inhibiting and anti-cancer effects 8, 9.

Signal transducer and activator of transcription (STAT) proteins are a family of cytoplasmic transcription factors,^10^ of which family member STAT3 is involved in many biological processes such as cell proliferation, survival, differentiation and angiogenesis 11-13. Many recent papers have pointed out that STAT3 becomes overexpressed in most human cancers and is associated with poor clinical prognosis such as tumour formation, metastasis and drug resistance 14, 15. Therefore, STAT3 is considered a potential therapeutic target for cancer treatment 16, 17.

Cluster of differentiation 47 (CD47) is a key anti-phagocytic signal for macrophages in the innate immune system 18. The innate immune system plays an important role in tumour surveillance, primarily via the phagocytic activity of macrophages 19, 20. In the early stages of tumour formation, macrophages actively infiltrate tumour tissues and phagocytose tumour cells; subsequently, their phagocytic ability is gradually inhibited by tumour-derived inhibitory signals 21. As the most studied anti-phagocytic signal in the tumour microenvironment (TME), CD47 has been shown to be overexpressed on the surface of multiple types of cancer cells. Binding of CD47 to its receptor signal regulatory protein α (SIRPα) on macrophages inhibits macrophage-mediated phagocytosis 22-24.

This study aimed to investigate the role of macrophage reprogramming by GMI treatment and overcome resistance in Osimertinib- and gefitinib-treated lung cancer cells.

## 2. Methods and Materials

### 2.1. Cell culture and chemicals

THP-1 cells were sourced from the Bioresource Collection and Research Center (BCRC, Taiwan). H1975 cells (L858R/T790M mutation), HCC827 cells (exon 19 deletion), gefitinib-resistant HCC827/GR cells and Tagrisso-resistant H1975/TR cells were provided by Dr. Ching-Chow Chen (National Taiwan University, Taiwan). HCC827/GR cells and H1975/TR cells were maintained with 2 µM gefitinib or Tagrisso. All cells were grown at 37 °C with 5% CO_2_ in RPMI-1640 medium (Gibco) supplemented with 10% foetal bovine serum (FBS), 100 µg/mL penicillin and 2 mmol/L L-glutamine. GMI^TM^, manufactured by Mycomagic Biotechnology Co., Ltd., (Taipei, Taiwan), was generated and ameliorated from *G. microsporum* and then stored at -20 °C until use. W1131 (HY-153190, MCE, USA) was prepared as a 3 mM stock solution in dimethyl sulphoxide (DMSO) for storage at -20 °C until use. DMSO and polybrene were acquired from Sigma-Aldrich (St. Louis, MO, USA).

### 2.2. Macrophages induce polarization

THP-1 cells were polarised into M0, M1 and M2 macrophages as previously described. The cells were cultured to 100 ng/mL PMA (19661, Caymen, Ann Arbor, MI, USA) for 72 h to obtain M0 macrophage. M1and M2 were obtained from 20 ng/mL IFN-γ (300-02, PeproTech, Rehovot, ISR, USA) plus 100 ng/mL LPS (L2280, Sigma-Aldrich, St. Louis, MO, USA) and 20 ng/mL IL-4 (200-04, PeproTech, Rehovot, ISR, USA) plus 20 ng/mL IL-13 (200-13, PeproTech, Rehovot, ISR, USA) for 48 h, respectively.

### 2.3. Plasmid constructions, cell transfection and virus infection

The pBabe-based vectors for the ectopic expression of N-terminal hemagglutinin epitope (HA)-tagged dominant-active STAT3 mutant (STAT3-C) were coined as pBabe-HA-STAT3-C, and they have been previously described 25, 26.

### 2.4. VZV-G pseudotyped lentivirus-shRNA system, cell transfection and virus infection

The knockdown of CD47 was accomplished using lentiviral-based RNAi reagents that were obtained from the National RNAi Core Facility located at the Institute of Molecular Biology/Genomic Research Center, Academia Sinica. Lentiviral infection of the HCC827 and H1975 cell lines was used to stably integrate and express short hairpin RNA (shRNA) targeting the CD47 mRNA sequences. The knockdown of CD47 was accomplished by lentiviral-specific short-hairpin RNA (shRNA) delivery, and the knockout of CD47 was accomplished by lentiviral delivery. The primer sequences were GCCTTGGTTTAATTGTGACTT, which were then used to infect HCC827 and H1975 cells in the presence of polybrene (8 μg/mL) to improve infection efficiency. Two days after infection, cells were subjected to positive selection by puromycin (2 μg/mL) for 48 h, followed by immunoblotting to confirm the ectopic expression of shCD47.

### 2.5. Clonogenicity assay

H1975, HCC827, HCC827/GR and H1975/TR (4 x 10^5^ /60 mm dish) were treated with GMI (0, 0.6 and 1.2 μM) for 24 h. The cells were trypsinised and replated at a density of 200 cells/60 mm-well plates to grow into colonies in drug-free media for 10-14 days. The cells were fixed with 95% ethanol and stained with a 20% Giemsa solution (1.09204.0500, Merck, DA, DE), and the numbers of colonies was scored as stated previously 27.

### 2.6. Flow cytometry

H1975, HCC827, HCC827/GR and H1975/TR (8 × 10^5^ cells/60 mm dish) were treated with GMI (0, 0.6 and 1.2 µM) for 24 h. Cells were washed with precooled PBS, trypsinised and incubated with a binding buffer containing annexin V-fluorescein isothiocyanate and propidium iodide (BioVision). Flow cytometry analysis was performed using FACS Calibur Flow Cytometer (BD Biosciences). At least 10,000 cells were analysed per sample and illustrated as a dot plot using CellQuest Pro software.

### 2.7. Western blot

Proteins were extracted from cells using RIPA buffer (RP05-10, Visual Protein, Taiwan) and supplemented with phosSTOP (04906845001, Roche) and protease inhibitors (04906837001, Roche). Protein lysates were determined with Bio-Rad Protein Assay Kit (500-0006, Bio-Rad, CA, USA). Proteins were separated by 10% SDS-PAGE and transferred to polyvinylidene fluoride (PVDF) membranes (IPVH00010, Millipore, USA) and incubated in 4 °C overnight with primary antibodies. Secondary antibodies were incubated in room temperature for 2 h. The PVDF membranes with target protein were exposed in Bio-rad Chemidoc MP system.

### 2.8 Antibodies

HA-tag (#3724), STAT3 (#9139) and phospho-STAT3 (Y705) (#9131) were purchased from Cell Signaling Technology (Boston, MA, USA). CD86 (A1199), CD206 (A11192), SIRP-alpha (A9001), Arginase 1 (ARG1; A22410) and CD163 (A23023) were purchased from ABclonal (USA, MA). β-actin was from Sigma (AC-40).

### 2.9. Quantitative RT-PCR

Total RNA was extracted from cultured cells using Rare-RNA (GRP02, GENEPURE TECHNOLOGY CO., Taichung Taiwan), and cDNA was synthesised by using High-Capacity cDNA Reverse Transcription Kits (4368813, Applied Biosystem). cDNA was used for quantitative RT-PCR by using SYBR Green (PT-GL-SQGLR-V3, Protech). The primer sequences for qRT-PCR analysis are listed in Table S1, and the primers were synthesised by Protech Technology Enterprise (Taiwan).

### 2.10. Phagocytosis assay *in vitro*

Macrophages were developed from THP-1 by PMA (100 ng/mL) and prepared for *in vitro* phagocytosis assay. Macrophages were cultured in serum-free medium for 2 h and co-cultured with tumour cells after treatment, which were labelled with CellTracker™ Green CMTPX Dye (C7025, Invitrogen™). CellTracker™ Red CMTPX Dye (C34552, Invitrogen™) was used to label macrophages. ImageXpress Pico was used to detect phagocytosis. The phagocytic index was calculated as the number of phagocytosed cells.

### 2.11. *In vivo* experiments

The *in vivo* mouse experiments were performed in accordance with the protocol approved by the Institution Animal Care and Use Committee (IACUC) of Chung Shan Medical University (protocol No. 2608). The five-week-old nude male mice (BALB/cAnN.Cg-Foxn1nu/CrlNarl) were obtained from BioLASCO (Taipei, Taiwan). To establish HCC827/GR tumour xenografts, mice were injected s.c., with 3x 10^ 6^ HCC827/GR cells (75μl) plus 75 μl Matrigel (BD Biosciences, 354234). Mice bearing HCC827/GR tumours were randomly separated into two independent groups (n = 3 for each group), including the control and GMI groups. Five days after cell implantation, mice in the control group were treated with 100 μl PBS by gavage once every day and served as controls. The GMI group was administered with 160 μg per mouse GMI diluted in 100 μl PBS by gavage once every day. Tumour sizes were measured every 3 days after 11 days of cell injection, and tumour volume was calculated by the formula 0.5x larger diameter (mm) x small diameter (mm) ^2^. At the end of the *in vivo* experiments, all mice were euthanized using CO2 and the subcutaneous HCC827/GR tumours were excised.

### 2.12. Statistical analysis

All data derived from three separate experiments were shown as the mean ± standard deviation. Student's t-test was used to analyse comparisons between two individual groups. One-way or two-way analysis of variance (ANOVA) was employed for comparisons involving multiple groups and/or conditions. p < 0.05 was considered as statistically significant.

## 3. Results

### 3.1. GMI inhibits STAT3/CD47 signaling and suppresses tumor growth in TKI-Resistant lung adenocarcinoma

**Figure 1:**
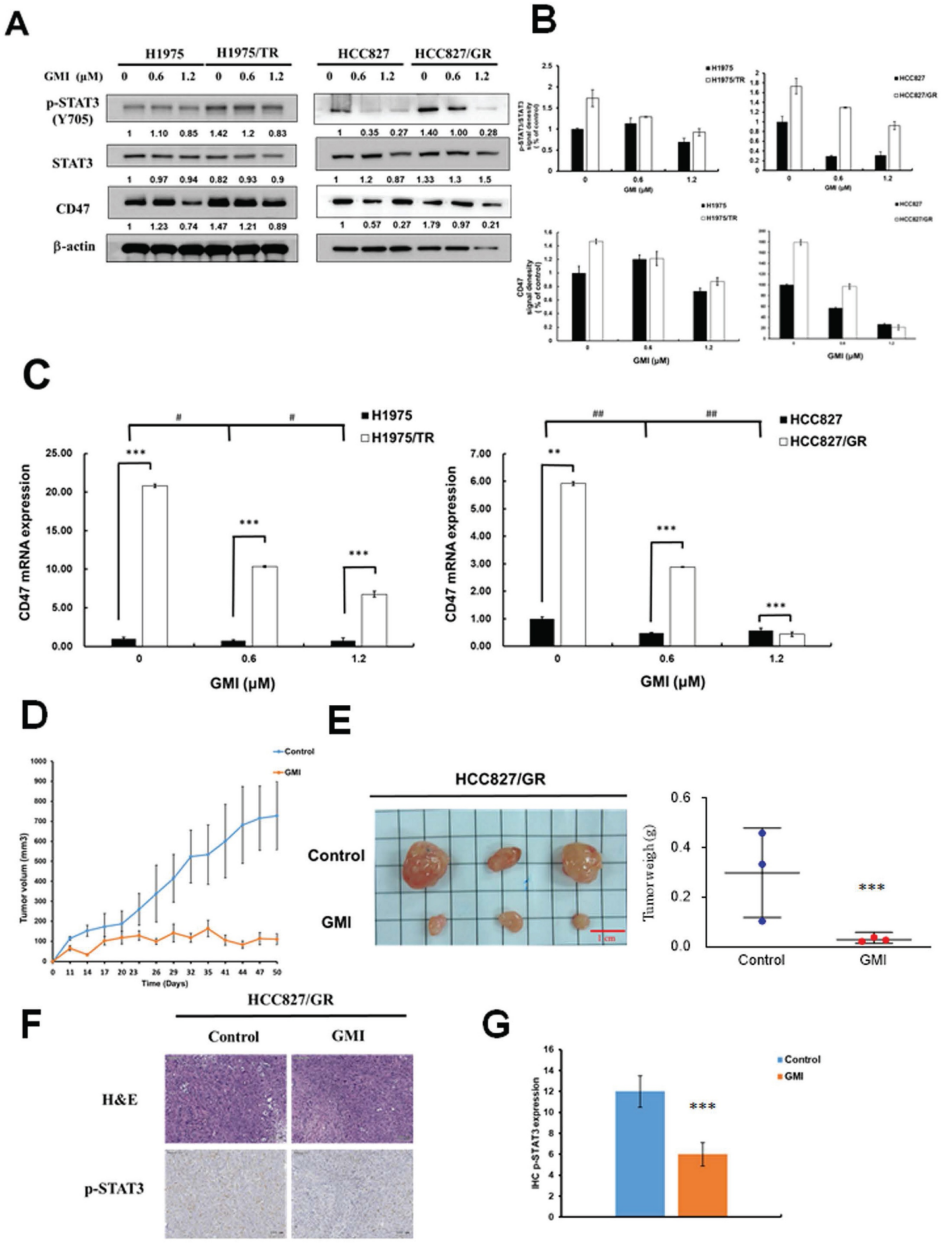
** Expression of STAT3 and CD47 in Parental and TKI-Resistant Lung Cancer Cells, and Tumor Growth *In Vivo*. (A)** After treatment of various concentrations of GMI (0, 0.6 and 1.2 μM) for 24 h, total cell lysates of H1975, H1975/TR, HCC827 and HCC827/GR cells (4 × 10^5^ cells in a 60 mm dish) were analysed by Western blot assay to detect the protein expression levels of p-STAT3(Y705) and CD47. **(B)** Protein densitometric quantifications of Western blot were performed using Image J software. **(C)** Quantitative RT analyses were performed to analyse CD47 gene expression. **(D)** Approximately 3 x10^ 6^ HCC827/GR cells were s.c., injected into studied mice to initiate tumour growth. Five days after cell implantation, the control group continued to receive sterilized PBS, whereas animals in GMI group received GMI protein (160 μg/mouse, N = 3, respectively). PBS and GMI protein were administered to mice by gavage once every day. Eleven days after cell transplantation, tumour sizes were measured every 3 days and the tumour volume was calculated. ***p < 0.001 with student's t-test. **(E)** The tumour weight of PBS group and GMI group were measured after mice sacrifice at day 50. Values are the mean ± SD on triplicate measurements. **(F)** H&E staining and IHC staining of p-STAT3 in tumor tissues. **(G)** Quantification of IHC staining were performed using Imagej software. *p < 0.05; **p < 0.01; ***p < 0.001. * compared with untreated cells.; ^#^ compared with parental and resistant cells.

Numerous studies have highlighted that aberrant activation of STAT3 is prevalent in lung cancer and various other malignancies. The transcription factor STAT3 regulates a multitude of genes associated with tumorigenesis, cell proliferation and metastasis, and its dysregulation is linked to poor prognosis. Therefore, we investigated whether GMI can reduce STAT3 and its downstream target CD47 in TKI-resistant cells. Western blot analysis was performed to assess the levels of phosphorylated STAT3 (p-STAT3, Tyr705) as a marker of activated STAT3 in lung adenocarcinoma cell lines treated with GMI. The results indicated that TKI-resistant cell lines HCC827 gefitinib-resistant (HCC827/GR) and H1975 Osimertinib and Tagrisso-resistant (H1975/TR) exhibited higher levels of p-STAT3 and CD47 compared with their parental cell lines. GMI also reduced p-STAT3 and CD47 among four cell lines (Figs. 1A and 1B). GMI could decrease CD47 mRNA expression in a dose-dependent manner (Fig. 1C). To evaluate the anti-cancer effect of GMI, an *in vivo* antitumor study was performed using a nude mice xenograft model subcutaneously inoculated with HCC827/GR cells. The average tumour volume of the treatment group (receiving GMI at 160 μg/mouse weight by gavage administration, N = 3) was statistically lower than that of the control group at day 23 (Fig. 1D). The mice were sacrificed at day 50. The tumour volume and the tumour weight of the GMI group is lower than the control group at day 50 (Fig. 1E). Immunohistochemical analysis revealed that the expression level of p-STAT3 was markedly reduced in the GMI-treated group compared to the control group (Fig. 1F and 1G).

### 3.2. Effect of Apoptosis and Cell Viability in STAT3 Overexpression Cells under GMI Treatment

**Figure 2:**
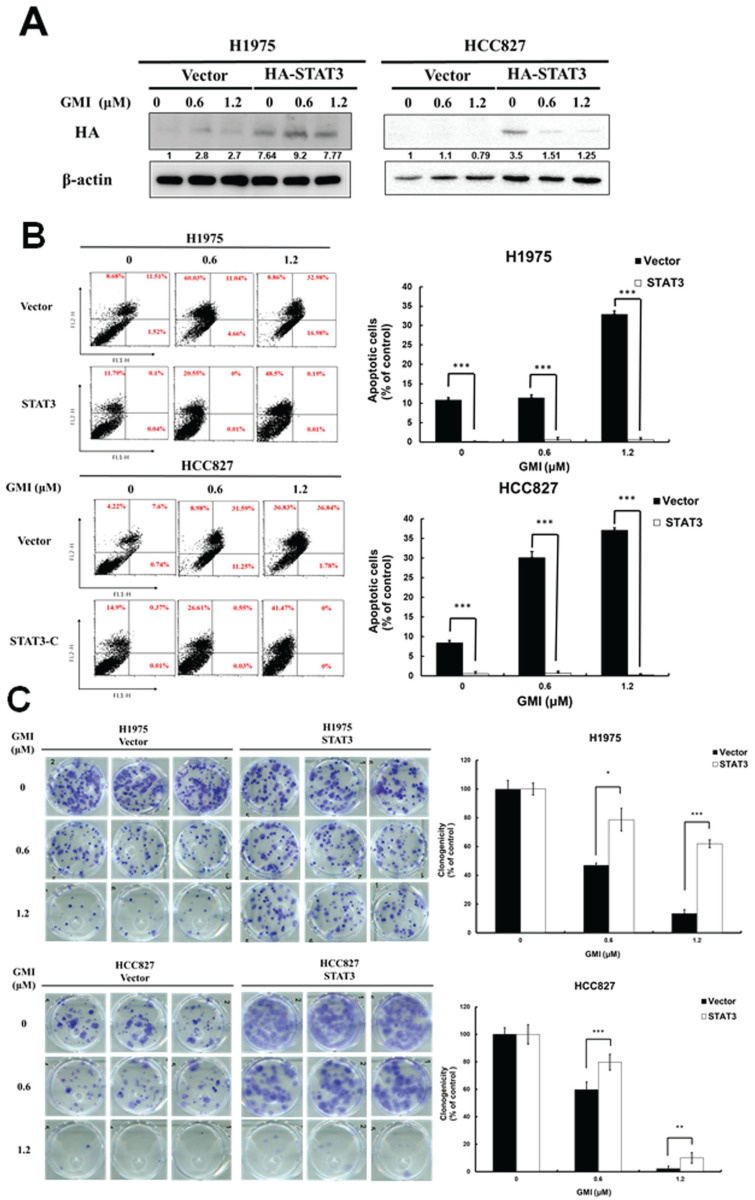
** Effect of GMI and overexpression of STAT3 in lung cancer cells. (A)** The vector pBabe-HA-STAT3-C (HA-STAT3), which continuously activates STAT3, infected into H1975 and HCC827 cells. After treatment of various concentrations of GMI (0, 0.6 and 1.2 μM) for 24 h, total cell lysates of H1975 and HCC827 and stable clone cells of STAT3-C (4 × 10^5^ cells of a 60 mm well) were analysed by Western blot assay to detect the protein expression of HA-taq. Beta-actin levels were used as equal loading control. **(B)** Left panel: Lung cancer stable clones of STAT3-C or its vector control cultured in a 6-well plate (4 × 10^5^ cells), and cells were treated with GMI (0, 0.6 and 1.2 μM) for 24 h, followed by three independent annexin V/PI staining experiments analysed by flow cytometry. Right panel: Values are the mean ± SD on triplicate measurements. **(C)** Colony formation assay. The number of colonies was counted under a dissecting microscope. We observed more than 50 cells in each colony. The data showed the relative colony number, and the number of cell lines without GMI treatment was set at 100%. # p < 0.05 compared with untreated cells; *p < 0.05 compared with overexpressed STAT3 or its vector control cell.

To clarify the role of STAT3 in GMI-induced apoptosis, we stably introduced a constitutively active STAT3 mutant (STAT3 A661C/N663C) with an N-terminal HA tag (HA-STAT3) into H1975 and HCC827 cell lines. Western blot analysis with an anti-HA antibody confirmed the sustained activation of STAT3 in these lung cancer cell lines (Fig. 2A). Flow cytometry was conducted following GMI treatment. The data showed 32.9%±0.7% and 37%±0.6% of apoptosis in H1975 and HCC827 cells, respectively. Overexpression of STAT3 ameliorated apoptosis to 0.5%±0.3% and 0.2%±0.2% (Fig. 2B). Cell viability was assessed via colony formation assay. The results showed that GMI treatment led to a significant decrease to 13%±2% and 2%±1% of un-infected cells (Vector). These values increased to 61%±2% and 9%±3% in constitutively STAT3-infected cells (Fig. 2C). Overall, these findings clearly confirmed that inhibition of STAT3 activation played an important role for GMI in inducing apoptosis in human lung adenocarcinoma cells.

### 3.3. CD47 mRNA and protein are regulated by STAT3

**Figure 3:**
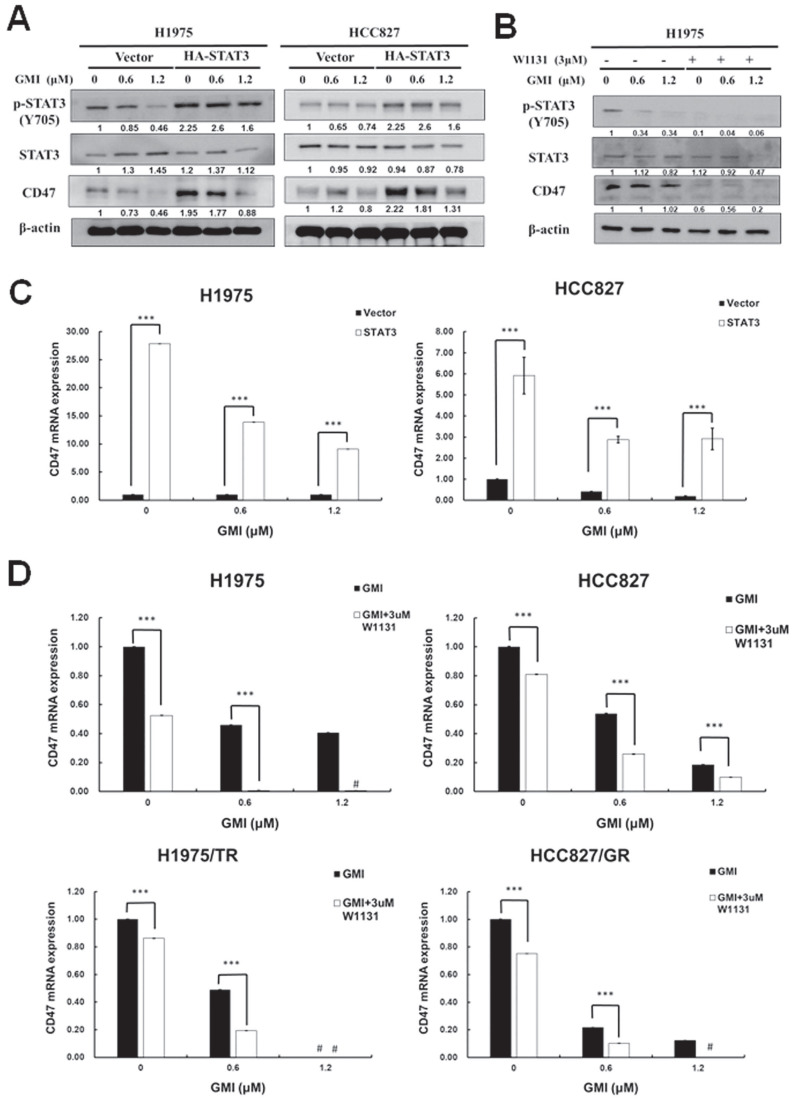
** Effect of GMI and overexpressed STAT3 on the expression of downstream CD47 protein. (A)** After treatment with various concentrations of GMI (0, 0.6 and 1.2 μM) in vector control and overexpressed STAT3 in H1975 and HCC827 cells. **(B)** Co-treatment with GMI and 3 μM p-STAT3 inhibitor W1131 for 24 h. Total cell lysates were analysed by Western blot assay to detect the protein expression levels of p-STAT3 and CD47. Beta-actin was used as equal loading control. **(C)** Quantitative RT-PCR assay to detect the mRNA expression of CD47 and **(D)** in parental (upper panel) and resistant (low panel) cells. Values are the mean ± SD on triplicate measurements. ns p > 0.05, *p < 0.05; **p < 0.01; ***p < 0.001. ^#^ p < 0.05 compared with untreated cells; *p < 0.05 compared with or without W1131. #undetermined: The CT value corresponding range of CD47 is over 40.

Activated STAT3 translocates to the nucleus to regulate gene expression. The above results indicated that GMI could inhibit STAT3 activation and CD47 expression. Phosphorylated STAT3 binds to the CD47 promoter and mediates CD47 expression. We observed that GMI affected CD47 protein levels. Western blot assay confirmed high levels of p-STAT3 and CD47 in constitutively active HA-STAT3 cells (Fig. 3A). When cells were treated with 3 μM STAT3 inhibitor W1131 in combination with GMI, STAT3 and CD47 protein levels were also reduced (Fig. 3B). The CD47 mRNA expression in overexpressd-STAT3 H1975 and HCC827 cells with GMI treatment was more pronounced than in H1975 and HCC827 vector cells. The difference in CD47 expression between the H1975/HCC827 vector and H1975/HCC827 overexpressed-STAT3 cells was more significant for cells treated with GMI than in other cells (Fig. 3C). The same results were obtained on quantitative PCR assay of mRNA of CD47. Treatment with 3 μM W1131 reduced CD47 mRNA. However, co-treatment with GMI significantly eliminated the expression of CD47 mRNA in H1975, HCC827, H1975/TR and HCC827/GR cells (Fig. 3D). Collectively, these results proved that inhibition of STAT3 activation regulated the downstream expression of CD47.

### 3.4. GMI Enhances Phagocytic Function of Macrophages Towards Lung Adenocarcinoma Cell Lines and Their Drug-Resistant Variants

**Figure 4:**
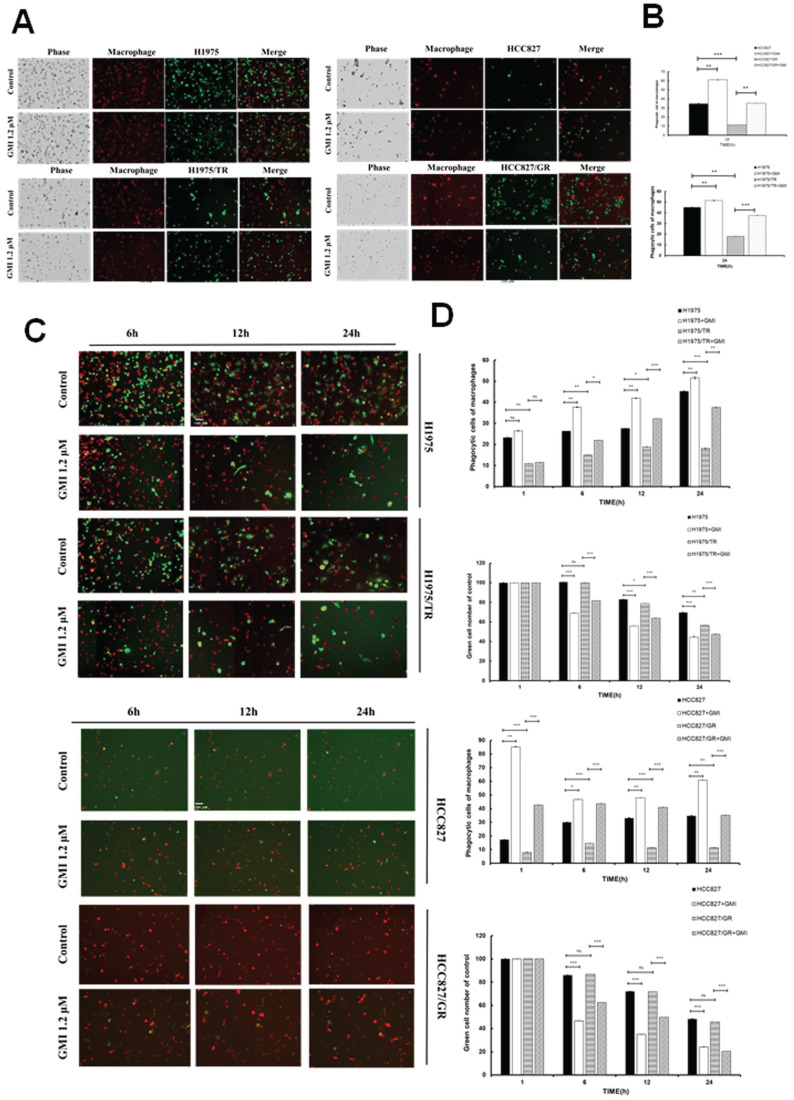
** Phagocytosis of sensitive and resistant lung cancer cells. (A)** Representative photography. H1975, H1975/TR, HCC827 and HCC827/GR cells (8× 10^3^ cells of a 96-well plate) were treated with 1.2 μM GMI for 24 h. **(B)** Values are the mean ± SD on triplicate measurements. **(C)** Fluorescence photography (left panel) and quantification (right panel) of macrophage (yellow)/tumour cells (green) treated with 1.2 μM GMI for different times (h) in H1975 and H1975/TR cells. (Macrophages: red; tumour cells: green; and yellow: phagocytosed cells. Scale bar, 200 μm. The phagocytic index was calculated as the ratio of the number phagocytosed cells of macrophages). **(D)** The above same conditions were shown in HCC827 and HCC827/GR. Values are the mean ± SD of triplicate measurements. ns p > 0.05, *p < 0.05; **p < 0.01; ***p < 0.001 compared with untreated cells.

Previous literature has established that CD47 interacts with the macrophage receptor SIRPα to initiate the 'Do Not Eat Me' signal, which inhibits phagocytosis and allows cells to evade engulfment. As observed in previous results, GMI reduces CD47 protein and mRNA levels. When the cells were damaged and phagocytosed, red macrophages progressively engulfed the damaged cells, resulting in yellow fluorescence due to the overlap of red (macrophage) and green (tumour cells) signals. The results indicated that the GMI promoter facilitated phagocytosis in four cells. Phagocytosis was activated to a lesser extent in Osimertinib-resistant cell (H1975/TR) and Gefitinib-resistant cells (HCC827/GR) (Fig. 4A and 4B). Furthermore, after treating cells with 0 or 1.2 μM GMI for 6, 12 and 24 h, we assessed phagocytic activity using ImageXpress Pico. The data revealed that phagocytic ability increased in a time-dependent manner to reach optimal for 24 h (Fig. 4C). Additionally, we observed a decrease in the number of green fluorescently labelled cancer cells over time, indicating enhanced phagocytosis. Phagocytic activity plateaued at 6 h in HCC827/GR (Fig. 4D).

### 3.5. STAT3 Influences Macrophage Phagocytic Activity

**Figure 5:**
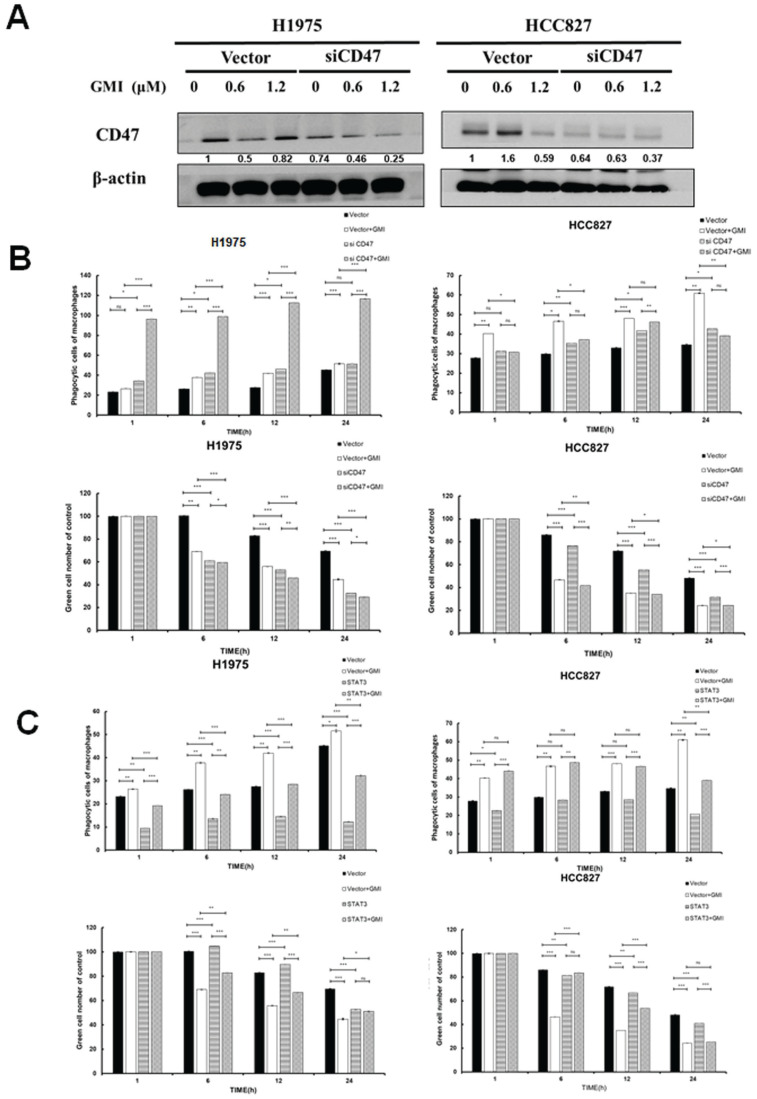
** Effect of phagocytosis on overexpressed STAT3 and silenced CD47. (A)** Western blot of CD47 expression in lung cancer cell lines stably transfected with control vector alone or siCD47 after treatment with GMI (0, 0.6 and 1.2 μM) for 24 h, followed by immunoblotting to assess CD47 silencing. **(B)** Quantification of phagocytosis of macrophage (yellow)/tumour cells (green) treated with 1.2 μM GMI for different times (h) in H1975 and HCC827 shLuc and siCD47 cells. **(C)** The above same conditions were shown in overexpressed STAT3 H1975 and HCC827 cells. Values are the mean ± SD of triplicate measurements. ns p > 0.05, *p < 0.05; **p < 0.01; ***p < 0.001.

We adapted from CD47 silencing and confirmed successful knockdown via Western blot analysis, which demonstrated a down-regulation of CD47 expression in HCC827 and H1975 cells (Fig. 5A). The results showed that silencing of CD47 significantly reduced the differences in phagocytosis between H1975 and HCC827 cells, as well as their drug-resistant mutant H1975/TR and HCC827/GR. Furthermore, GMI treatment enhanced phagocytosis in these cell lines (Figs. 5B and S2A). Given that STAT3 mediates CD47 expression, we further explored this relationship by using stable cell lines with constitutively active STAT3. After treating these cells with different concentrations of GMI, we assessed phagocytic activity. The phagocytic activity was restored in cells with STAT3 overexpression (Figs. 5C and S2B). Overall, these findings demonstrated that up-regulation of STAT3 and CD47 contributed to the evasion of tumour cells from macrophage-mediated phagocytosis.

### 3.6. Effects of GMI on Macrophage Polarization to M0, M1 and M2 Phenotypes

**Figure 6:**
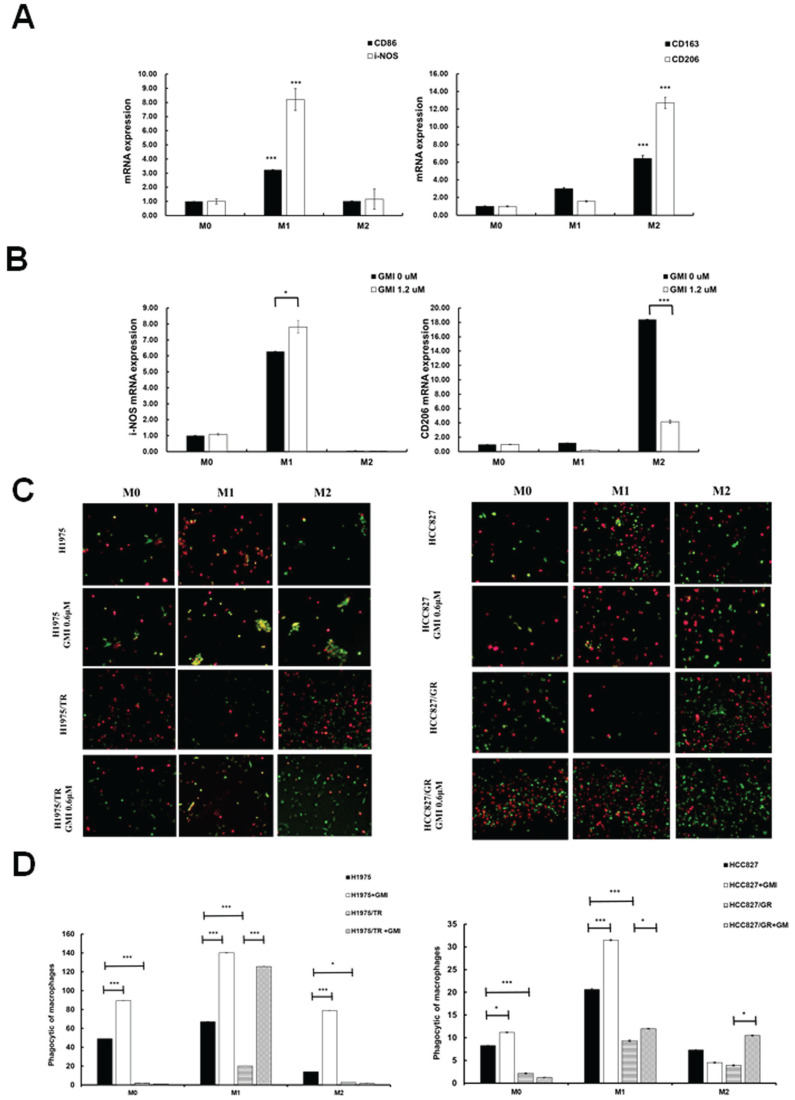
** GMI triggers macrophage polarization. (A)** Cell lysates of M0, M1 and M2 cells (4 × 10^5^ cells of a 60 mm well) were analysed by quantitative RT-PCR assay to detect the mRNA expression levels of CD86 and CD206. **(B)** Polarised M1 or M2 cell (4 × 10^5^ cells of a 60 mm well) treated with 1.2 μM GMI for 24 h. The cDNA was analysed by quantitative RT-PCR assay to detect the mRNA expression of I-NOS and CD206. **(C)** Fluorescent images of H1975, H1975/TR, HCC827 and HCC827/GR cells (8× 10^3^ cells of a 96-well plate) after treatment with 1.2 μM GMI for 24 h, followed by co-culture with M0, M1 and M2 for 6 h. They cells were assessed by fluorescence microscopy and **(D)** quantitatively analysed for phagocytosis. Values are the mean ± SD of triplicate measurements. ns p > 0.05, *p < 0.05; **p < 0.01; ***p < 0.001 compared with untreated cells.

We aimed to investigate whether GMI influences macrophage differentiation and its impact on M1 (pro-inflammatory and anti-tumour) and M2 (anti-inflammatory and pro-tumour) macrophages in response to cancer cells. To this end, we established a macrophage polarization model using THP-1 cells. Initially, we differentiated monocytes into macrophages by treating them with phorbol 12-myristate 13-acetate (PMA). Once differentiated into M0 macrophages, we further polarised them by incubating with IL-4 and IL-13 to obtain M2 macrophages, or with IFN-γ and LPS to activate classical M1 macrophages. Western blot analysis was then performed to assess the protein levels of CD206 (an M2 marker) and CD86 (an M1 marker). The results showed the successful polarisation of macrophages, with GMI effectively reducing CD206 protein levels in M2 macrophages and increasing CD86 protein levels in M1 macrophages (Fig. S3A). To further validate these findings, we analysed mRNA expression of M0, M1 and M2 macrophages by RT-PCR. Measurement of several classic M1 and M2 markers confirmed the successful polarisation of macrophages, as evidenced by significant increases in M1 markers (CD86 and i-NOS) and M2 markers (CD163 and CD206; Fig. 6A). Furthermore, GMI treatment led to an increase in i-NOS mRNA levels in M1 macrophages and a decrease in CD206 mRNA levels in M2 macrophages, confirming that GMI enhanced pro-inflammatory and anti-tumour M1 macrophages while reducing the anti-inflammatory and pro-tumour M2 macrophages (Fig. 6B). Specifically, GMI increased the phagocytic ability of M1 macrophages towards H1975, H1975/TR, HCC827 and HCC827/GR cells (Figs. 6C and 6D).

## 4. Discussion

In this study, the results showed that up-regulation of STAT3 and CD47 helped tumour cells escape phagocytosis by macrophages, and GMI could participate in the inhibition of the STAT3-CD47 signalling axis. These results support GMI as a potential drug for cell-targeted treatment of lung cancer.

We used GMI to treat H1975 and HCC827 cells and its drug-resistant strains H1975/TR and HCC827/GR; they all had exhibited remarkable ability to inhibit cell survival. In addition, STAT3 is associated with many cancers 28, 29. A large amount of evidence has been published in many papers showing that activation of STAT3 plays a key role in the process of malignant transformation 30, 31. Therefore, STAT3 is an extremely important oncogenic factor and is crucial to tumour progression and the formation of the TME 32. Evidence from other laboratories suggested that STAT3 is selectively active in response to TKI target drugs 33. GMI can also regulate p-STAT3 (STAT3 activation state) in lung cancer cells. These results were consistent with the discovery of the mechanism of GMI in oral cancer 34, 35. GMI can inhibit STAT3 and the growth of cancer cells. Currently, the STAT3 inhibitor AZD9150 is used in clinical medical research in the treatment of lung cancer 36, 37.

We have several inferences about how GMI inhibits STAT3. Among them, EGFR mutations are a key therapeutic target and are common in NSCLC 38. Significant progress has been made in the clinical management of EGFR mutations through targeted therapy. These TKIs in cancer cells block EGFR tyrosine kinase phosphorylation and downstream signalling pathways such as MAPK and AKT 39, 40. This inhibits tumour proliferation, promotes apoptosis and resists angiogenesis, ultimately achieving anti-tumour effects.

We observed a positive correlation between STAT3 and CD47 expression in lung cancer. Therefore, we screened CD47 at four recognised checkpoints in phagocytic cells in EGFR-TKI-resistant NSCLC. In this study, we found that phosphorylated STAT3 and CD47 were significantly up-regulated in drug-resistant cells. This phenomenon may be the reason why drug-resistant cells are resistant. Other studies also mentioned that a large number of STAT3 expressions are related to drug resistance. The positive correlation between STAT3 and CD47 expression in lung cancer was consistent with previous findings, as Kang *et al.* (2023) demonstrated that the α5-nAChR/STAT3/CD47 axis promotes lung adenocarcinoma progression and immune evasion, supporting a role for STAT3 as a direct regulator of CD47 expression. 41 Shrestha *et al.* (2025) reported that combined inhibition of STAT3 and CD47 effectively suppressed lung metastasis in osteosarcoma, highlighting the therapeutic relevance of targeting this pathway 42. In clinical studies, the SIRPα receptor is expressed on macrophages and is an inhibitory immune receptor. After binding to the CD47 protein, it sends a 'do not eat me' signal. Given that CD47 is often overexpressed in cancer cells to avoid clearance by macrophages, treatments targeting CD47/SIRPα have been actively investigated 43, 44. We found that CD47 was significantly up-regulated in drug-resistant cells, which further demonstrated that the up-regulation of CD47 helped tumour cells escape phagocytosis by macrophages. GMI inhibited STAT3, reduced macrophage M2 polarization and SIRPα levels and enhanced their immune and anti-tumour functions.

The clinical application of EGFR-TKIs faces many challenges. Although numerous highly selective drugs and combination treatment strategies targeting EGFR protein mutation sites have been developed, acquired TKI resistance has not been effectively solved. The TME is a comprehensive system formed by the interaction of tumour cells with surrounding tissues and immune cells. Cells in the TME are reprogrammed to adapt to the environment during drug treatment. In recent years, the role of the TME in acquired TKI resistance has attracted increasing attention 45. Macrophages are one of the main components of the TME and may be closely related to tumour occurrence and drug resistance. In other papers, down-regulation of macrophage phagocytosis and up-regulation of macrophage M2 polarization were found to be associated with NSCLC drug resistance, indicating that TAM plays an important role in drug resistance 46.

In conclusion, our data proved that GMI enhanced phagocytosis, which influenced TAMs and modulated the STAT3-CD47-SIRPα signalling axis involved in EGFR-TKI resistance. Thus, GMI represents a novel therapeutic strategy to overcome EGFR-TKI resistance in lung cancer. The observed acquired resistance to TKIs underscores the potential of GMI as a targeted therapeutic agent for lung cancer cell lines and drug-resistant variants. The findings of this study warrant further investigation into the development of GMI as an anti-cancer agent.

## Supplementary Material

Supplementary figures and table.
