# Supplementary material for: Ganoderma microsporum Immunomodulatory Protein (GMI) Enhances Phagocytosis by Suppressing STAT3/CD47 Signaling in EGFR-Mutant NSCLC Resistant to Gefitinib and Osimertinib
Source: J Cancer. 2026 Jan 1;17(1):86–98. doi: 10.7150/jca.124363 (PMC12719595; doi:10.7150/jca.124363)
Supplement: Supplementary file 1 — Supplementary figures and table. [file jcav17p0086s1.pdf]

Gene	Primer sequence
GADPH	F: 5'- CCATGGGGAAGGTGAAGGTC-3'
	R: 5'- GCGCCCAATACGACCAAATC-3'
iNOS	F: 5'- AGGGACAAGCCTACCCCTC -3'
	R: 5'- CTCATCTCCCGTCAGTTGGT -3'
CD86	F: 5'-ACCTGCGACAGTAAACGAGG-3'
	R: 5'-TGTCTCCGCTTCATGCCATT-3'
CD206	F: 5'-TCTTCTCGAACCCCGAGTGA-3'
	R: 5'-TATCTCTCAGCTCCACGCCA-3'
CD163	F: 5'- AGCATGGAATGACGACAGTG -3'
	R: 5'- GATGTGGCCCCTGGTAGC -3'
CD47	F: 5'- TCATCTGGACAAGCAGTGACC -3'
	R: 5'- AGGTAGTTCTGGGATGACCAA -3'

Table S1. The sequences of primers for the qRT-PCR analysis

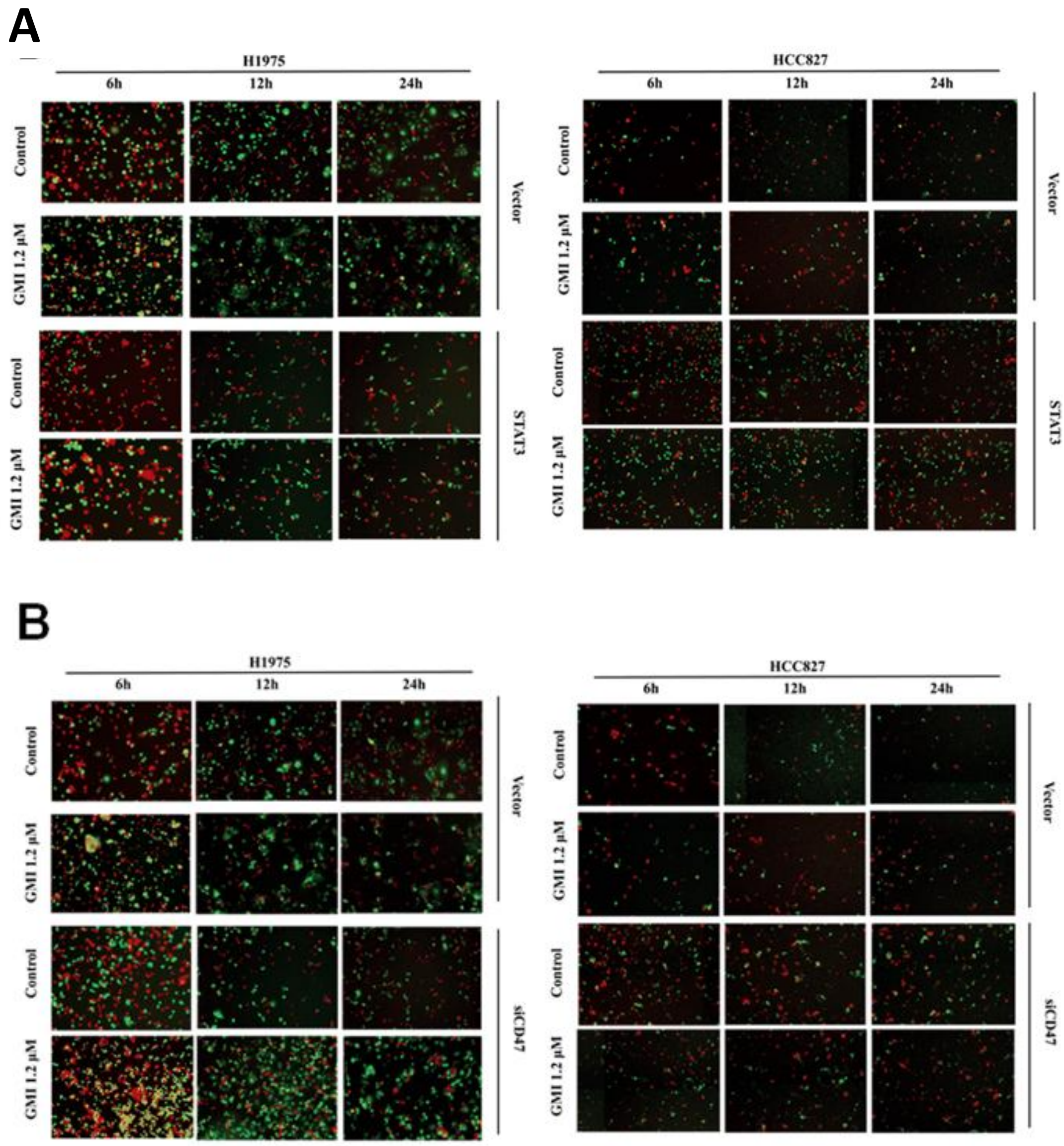

Figure S2. Effect of phagocytosis on overexpressed STAT3 and silenced CD47

(A) Fluorescent images of phagocytosis of macrophage (yellow)/tumour cells (green) treated with 1.2 μM GMI for different times (h) in H1975 and HCC827 shLuc and siCD47 cells. (B) The above same conditions were shown in overexpressed STAT3 H1975 and HCC827 cells.

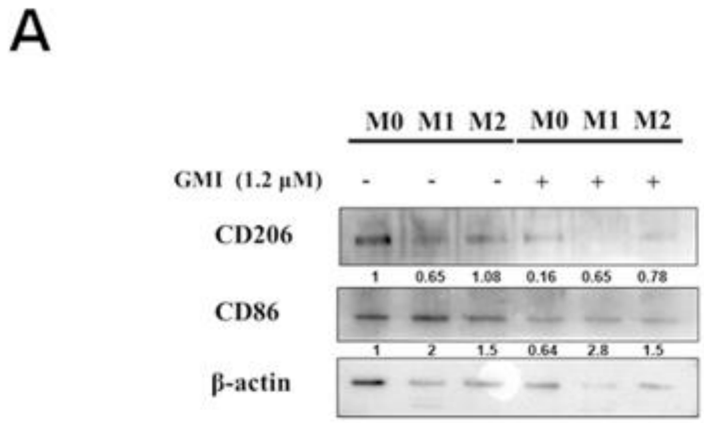

Figure S3. GMI triggers macrophage polarisation.

(A) Cell lysates of M0, M1 and M2 cells (4×10^5 cells of a 60 mm well) were analysed by Western blot assay to detect the protein expression of CD86 (M1 marker) and CD206 (M2 marker).
